# Implementation Science: Helping to Accelerate Progress Toward Achieving the 90–90–90 Goal

**DOI:** 10.1007/s10461-019-02649-8

**Published:** 2019-09-11

**Authors:** Waimar Tun, Vivian Go, Aisha Yansaneh

**Affiliations:** 1 HIV and AIDS Program, Population Council, 4301 Connecticut Avenue, NW, Suite 280, Washington, DC 20008 USA; 2 Gillings School of Global Public Health, University of North Carolina, Chapel Hill, NC USA; 3 United States Agency for International Development (USAID), Washington, DC USA

In 2014, the Joint United Nations Programme on HIV/AIDS (UNAIDS) announced the 90–90–90 targets to diagnose 90% of all HIV-positive persons, to provide antiretroviral therapy (ART) to 90% of those diagnosed, and to achieve viral suppression for 90% of those on ART by 2020 [1]. Significant progress has been made toward achieving this target. In 2018, 79% of people living with HIV (PLHIV) knew their status; 78% of those who knew their status were on ART; and, among people on ART, 86% were virally suppressed [2]. While established evidence-based interventions (EBIs), including voluntary medical male circumcision (VMMC) [3, 4], ART to treat and prevent HIV infection [5–7], HIV testing services [8, 9], and prevention of mother-to-child transmission [10], have facilitated progress, they have not reached their full potential due to implementation barriers such as HIV-related stigma, mental health co-morbidities, overburdened health facilities and providers, and under-trained staff. We know what interventions work, but we need better evidence on how to implement them with fidelity and overcome real-world barriers, as well as how to ensure utilization and translation of the evidence into policy and practice.

To accelerate progress toward the 90–90–90 targets, innovative and more refined implementation strategies to access hard to reach populations are imperative. Implementation science, “a multidisciplinary scientific field that seeks generalizable knowledge about the magnitude of, determinants of, and strategies to close the gap between evidence and routine practice for health in real-world settings,” can help identify strategies to address these barriers [11]. The United States President’s Emergency Plan for AIDS Relief (PEPFAR) adopted an implementation science framework in 2011 as its focus shifted from providing emergency relief to developing sustainable strategies [12]. With the increased focus on controlling the HIV epidemic through investing in EBIs in geographic areas and populations with the highest burden to achieve maximum impact, implementation science can play a pivotal role in reaching the 90–90–90 targets.

In 2014, the United States Agency for International Development made an investment to maximize the efficiency and impact of programs and policies by supporting implementation science research through Project SOAR—Supporting Operational AIDS Research. The objectives of the six-year initiative are to: (i) conduct high-quality implementation science research to improve HIV program implementation, (ii) strengthen capacity among local institutions to conduct high-quality implementation science research, and (iii) promote use of study findings to make informed program and policy decisions. Since its inception, Project SOAR has conducted 70 activities in 21 countries. This supplement features findings from select studies gleaned from the third and fourth years of the project. The articles cover all three pillars of the project: research, capacity strengthening, and research utilization.

SOAR is generating findings to improve the efficiency and effectiveness of HIV programs by addressing emerging issues, evidence gaps, and country needs. This special issue takes a broad perspective and includes papers examining the implementation strategies in new contexts [community-based ART distribution and HIV self-testing (HIVST)] and exploring barriers along the HIV testing and treatment cascade (gender and depression), and methodological topics [modeling, resilience scale development, and mapping and time-location sampling (TLS) to reach a vulnerable population]. Additionally, a case study and commentary on two cross-cutting issues addressed by Project SOAR are included, namely that of capacity strengthening of local researchers and organizations and the use of a systematic research utilization process to translate study findings into action.

The articles in this special issue address critical barriers and dilemmas faced by those implementing HIV programs by framing problems through an implementation science lens and identifying practical solutions for enhancing the effectiveness of EBIs. The papers are organized by the following topics: linkage to care, factors that affect the HIV care cascade, methodologies to improve implementation science research and HIV programming, and ensuring that research makes a difference.

## Linkage to Care

Successfully linking to the HIV care continuum involves a complex series of processes that extend beyond simply getting tested and referred to treatment. Herce et al. propose a new conceptual framework that appreciates the nuances of linking to care (LTC), a process that is often over-simplified [13]. From time of testing HIV positive, multiple steps must occur. The LTC framework includes appropriately educating or counseling a patient, facilitating transfer to the care and treatment department, investigating for co-morbid infections, assessing safety of the planned ART regimen during clinical evaluation, initiating ART and dispensing other medications, providing early support and completing a first follow-up visit; all within a reasonable timeframe of 1 month. The framework, which draws from the social ecological model and the conceptual model of implementation research, can help to specify barriers to linkage and facilitate the development of targeted implementation approaches to address these barriers.

Low use of HIV testing and treatment services can be explained by a combination of behavioral, social, and health systems factors. Among key populations, HIV stigma and criminalization are a major barrier to HIV testing. HIVST offers increased convenience, privacy, and autonomy, and can normalize regular testing. Lyons et al. conducted a study to assess venue- and social network-based distribution on uptake of HIVST in Senegal [14]. HIVST reached a high proportion of first-time testers, and the majority of self-reported reactive results were among first time testers, suggesting that HIVST can be effective in increasing HIV diagnoses. As HIVST is scaled up globally, this study provides important implementation insights on penetration, acceptability, and use of HIVST among populations at high risk for HIV.

Despite the rapid scale up of ART since 2000, gaps in ART uptake and retention persist. For female sex workers, distance to clinics, cost, and sex worker-related stigma are key barriers to HIV care and treatment. Tun et al. present findings from an implementation science study of a community-based ART distribution program for female sex workers living with HIV in Tanzania [15]. Those who received community-based ART were significantly more likely to have initiated and adhered to ART than those who received facility-based ART. Community-based ART could provide a comprehensive alternative to facility-based care for female sex workers who often have mobile, unstable lifestyles and who face high levels of sex-worker related stigma.

## Factors that Affect the HIV Care Cascade

Kulisewa et al. and Pulerwitz et al. highlight two factors that affect HIV testing, and ART uptake and adherence: mental health and gender norms, respectively. In order to achieve the UNAIDS 90–90–90 targets, Kulisewa et al. points out that it may be necessary to address mental health disorders, such as depression, that are pervasive among PLHIV [16]. Several studies have demonstrated that depressed individuals are less likely to be tested, initiate ART, and adhere to ART compared to their non-depressed counterparts [17–19]. The lack of mental health infrastructure and human resources to address the burden of mental health disorders in sub-Saharan Africa have warranted novel strategies, such as task-shifting approaches. Enhancing the capacity of primary care providers and lay health workers to effectively diagnose and manage depression has been shown to be a cost-effective method for scaling up mental health services [20, 21]. Kulisewa et al. describes findings that demonstrate the feasibility and acceptability of integrating depression screening into HIV clinics in Malawi.

Pulerwitz et al. assessed the influence of endorsement of inequitable gender norms on HIV testing and uptake of ART in South Africa [22]. Using data from a 2014 population survey in rural South Africa, Pulerwitz et al. measured attitudes toward gender norms using the validated GEM Scale. They found that participants who more strongly endorsed inequitable gender norms were less likely to be engaged in HIV treatment; this was particularly true for women. One of the first studies to quantitatively explore the role of gender norms in the HIV continuum of care, this study complements the existing qualitative literature that has explored this topic.

## Methodologies to Improve Implementation Science Research and HIV Programming

Implementation science is about understanding how interventions can be implemented effectively and efficiently. However, researchers need the appropriate tools to help measure the impact of these interventions. In this era of effective HIV treatment, with PLHIV living longer and healthier lives, methods to measure resilience in the context of living with HIV is critical, as resilience has been shown to be associated with important HIV-related treatment outcomes. Gottert et al. developed and validated a PLHIV resilience scale [23]. Previous resilience scales have not been specific to living with HIV. Only one scale (PozQoL) measures resilience in the context of HIV [24]; however, the authors indicate it may be too broad to be useful as a brief tool. The 10-item PLHIV-specific resilience scale by Gottert et al. was found to have satisfactory psychometric properties and now forms part of the PLHIV Stigma Index 2.0 [25]. This is an important addition to the toolbox to track important indicators among PLHIV to ultimately inform programs and policies.

Innovative methods are also needed for EBIs to reach vulnerable populations to ensure that we can attain the 90–90–90 goals. Development of new strategies and more intentional recruitment efforts are thus needed to reach deeper into at-risk communities that are missed by traditional methods of recruitment. Wang et al. describes a targeted approach to identifying and accessing adolescent girls and young women (AGYW) at increased risk for HIV infection [26]. They report on the effectiveness of using a community-informed venue mapping and TLS in reaching vulnerable AGYW in Addis Ababa. While venue mapping and TLS are not new methodologies, applying them to reach this population is new. Traditionally, school- and household-based approaches have been used to reach vulnerable communities; however, these methods may not identify the most vulnerable AGYW such as out-of-school youth and those without housing. The authors report the effectiveness of venue mapping and TLS in identifying highly vulnerable AGYW.

The article by Stegman et al. highlights the importance of using the Decision Makers Program Planning Tool (DMPPT), to help programmers prioritize certain subsets of the population for VMMC [27]. In this case, the DMPPT was a useful tool to help understand the potential impact of circumcising different age groups on HIV in Namibia. They demonstrate that focusing VMMC efforts on specific age groups can lead to more efficient use of program resources. Modeling exercises such as this are critical for VMMC strategy formulations and target setting for the country, given resource and financial constraints.

## Ensuring that Research Makes a Difference

One of the three pillars of Project SOAR—strengthening capacity of local stakeholders to conduct research—seeks to ensure local engagement in the entire research process and ultimately local ownership of the research. With the shift toward more local engagement and ownership of the HIV response and donors providing funds directly to local nongovernmental organizations, capacity strengthening activities are of vital importance. Often capacity strengthening activities around research are conducted to increase an individual’s capacity in specific skills such as protocol writing or data analysis; however, there is little attention to the institutional contexts within which individuals operate. The implementation science capacity strengthening workshop described in the manuscript by Kalbarczyk et al. emphasizes the importance of institutional capacity (e.g., physical infrastructures, record keeping and accountability structures, systems, and roles within the structure) that support an individual’s successful implementation of research [28]. An important outcome of the workshop was participants’ understanding of their own and their organization’s needs at the various levels of capacity strengthening and appreciation in engaging local stakeholders to make their research relevant to the local context.

In addition to strengthening the capacity of local stakeholders, it is crucial for local stakeholders to be involved in the research process to ensure that research findings have an impact on policies and programs. The article by Kalibala et al. presents a research utilization model to ensure the research is locally relevant, owned, and utilized [29]. The research utilization model moves researchers away from the traditional, passive model whereby researchers disseminate final findings with stakeholders only at the end. The authors provide examples of demonstrating that when stakeholders are involved in the entire process, there is impact on programming and policies. Researchers must “start with the end in mind” in order to facilitate the uptake of the research findings. We strongly urge researchers to utilize an active research utilization model and design and implement studies with the holistic view toward translating results into impact. Equally important, donors should prioritize this active process in their funding of implementation science, and governments should commit to engaging in the research process to ensure that implementation science makes a difference. Implementation science research cannot make a difference if results are not used to inform policy and practice.

Taken together, this collection of articles reflects the importance of conducting implementation science studies to understand how interventions can be implemented in real-world settings. Project SOAR’s implementation science agenda has helped to identify solutions to some of the key barriers along the HIV care cascade and has provided important tools that can be used in implementation science studies. Implementation science research is needed to offer useful insights into how EBIs can be improved and refined for greater efficiency and effectiveness in different contexts and in real-world settings as we near the 90–90–90 targets. Despite the recognition in the scientific community that implementation science can play a pivotal role in reaching the 90–90–90 target, implementation science studies remain woefully underfunded [30–32]. Investments in implementation science must continue to be an essential component of the global and national HIV responses.
